# Relationship between S100A4 protein expression and pre-operative serum CA19.9 levels in pancreatic carcinoma and its prognostic significance

**DOI:** 10.1186/s12957-019-1707-4

**Published:** 2019-09-16

**Authors:** Fuxin Jia, Mengmeng Liu, Xiao Li, Fen Zhang, Shuqiang Yue, Jiangwei Liu

**Affiliations:** 1 Department of Hepato-pancreatico-biliary Surgery, Luo Yang Central Hospital Affiliated to Zheng Zhou University, No. 288 Zhongzhou Middle Road, Luo yang, 471000 Henan Province China; 2 Infectious Disease Prevention and Control Institute, Luo Yang Center for Disease Control and Prevention, No. 9 Zhenghe Road, Luo yang, 471000 Henan Province China; 3 Department of Hepato-Pancreatico-Biliary Surgery, Xijing Hospital, Air Force Medical University, No.15 Changle West Road, Xi’an, 710032 Shanxi Province China; 4 Key Laboratory of Special Environmental Medicine of Xinjiang, General Hospital of Xinjiang Military Command of the PLA, NO. 359 Youhao North Road, Urumuqi, 830000 Xinjiang Uygur Autonomous Region China

**Keywords:** S100A4, CA19.9, Pancreatic carcinoma, Correlation, Cancer biomarkers, Prognosis

## Abstract

**Background:**

Pancreatic carcinoma (PC) is one of the most lethal malignancies, and its poor prognosis is strongly associated with invasion and metastasis. CA19.9 is considered to be the most sensitive serum marker for PC in clinical practice; however, the detection of CA19.9 in PC has a certain false positive and false negative rate. The expression of the calcium-binding protein S100A4 has been reported to be associated with poor prognosis in various cancers. This study aimed to investigate the relationship between S100A4 and CA19.9 and its prognostic significance in PC.

**Methods:**

We performed immunohistochemical staining for S100A4 in formalin-fixed, paraffin-embedded blocks of 128 PC tissues. The levels of S100A4 expression and pre-operative serum CA19.9 were correlated with clinicopathological parameters. The possible correlation between S100A4 protein expression and pre-operative serum CA19.9 levels were evaluated using the chi-square test and Spearman correlation. Survival was assessed by Kaplan–Meier analysis together with a single variable or multivariate Cox analysis.

**Results:**

A significant positive correlation between S100A4 expression and pre-operative serum CA19.9 level was observed in PC tissues (*ρ* = 0.202, *P* = 0.022). The co-expression of both proteins correlated significantly with tumor differentiation (*ρ* = − 0.280, *P* = 0.001), TNM stage (*ρ* = − 0.389, *P* = 0.000), and lymph node metastasis (*ρ* = 0.254, *P* = 0.008). Upregulation of S100A4 was identified as a significant, independent predictor of poor overall survival (*P* = 0.000). Moreover, higher serum CA19.9 levels (≥ 35 U/mL) were also recognized as an independent predictor of inferior overall survival (*P* = 0.001). Additionally, upregulation of S100A4 and higher pre-operative serum CA19.9 levels (≥ 35 U/mL) in patients with PC contributed to a significant decrease in overall survival (*P* = 0.000).

**Conclusions:**

The expression levels of S100A4 in PC tissues were positively correlated with pre-operative serum CA19.9 levels. S100A4 expression and pre-operative serum CA19.9 levels were significant, independent prognostic factors for the overall survival of patients with PC. S100A4 expression/pre-operative serum CA19.9 levels may prove useful as dual prognostic biomarkers for PC. Analysis of CA19.9 in combination with S100A4 can better predict the prognosis of PC.

## Background

Pancreatic carcinoma (PC) is one of the most malignant gastrointestinal tumors, with a mortality rate that nearly equals its incidence rate [1]. Worldwide, it is reported to lead to an estimated 227,000 deaths annually, making it the fourth leading cause of cancer-related mortality in some developed countries [2–6]. According to the statistics of the American Cancer Society, the number of individuals with newly diagnosed PC in the USA in 2019 is estimated to be 56,770, with 45,750 deaths due to PC [7]. By 2030, PC is expected to be the second leading cause of cancer-related mortality in the USA [8]. In the same period, the latest data released by the Chinese National Cancer Center also showed that the incidence of PC in China increased to become the tenth highest occurring malignant tumor and has the sixth highest cancer-related mortality rate; in some large cities, the incidence of and mortality associated with PC has risen to rank seventh and fifth, respectively [9]. It is difficult to diagnose PC at an early stage, with the vast majority of cases found to have metastasized tumors at the first diagnosis, and 10.3% of PC cases have localized tumors at the time of diagnosis [10]. Moreover, due to its resistance to radiation therapy and chemotherapeutic agents, aggressive invasion, as well as metastasis to other organs, the early diagnosis of PC is critical but difficult and the effects of standard therapy are limited [11]. The only treatment for PC is surgical resection. Unfortunately, owing to late presentation, only 15 to 20% of the diagnosed patients are eligible for pancreatectomy. However, the prognosis remains very poor even after the complete resection. The 5-year survival rate after pancreaticoduodenectomy or Whipple surgery was approximately 21% for negative marginectomy (R0) resection and 11% for microscopically positive marginectomy (R1) resection. Moreover, up to 71% of patients who have undergone curative R0 resection show recurrence of the disease [12]. Therefore, to provide optimal opportunity for the improvement in the prognosis of PC and increase the survival rates, it is crucial to come up with a method with high sensitivity and specificity to help risk-stratify patients based on their outcomes and to identify subpopulations that may benefit from specific therapies.

CA19.9 is considered to be the most sensitive serum marker for PC in clinical practice [13, 14]. Although CA19.9 has a high diagnostic value for PC, its sensitivity and specificity are not satisfactory. CA19.9 belongs to the Lewis group of blood antigens (including Lewis-a/b), and individuals with Lewis-negative phenotype cannot synthesize CA19.9. Approximately 5–10% of the population is Lewis negative, which may lead to false-negative results of CA19.9 in PC patients [15]. In addition, the CA19.9 levels were observed to be close to normal in patients with partially poorly differentiated PC, and changes in CA19.9 levels have also been known to be indicative of other diseases. Therefore, the diagnosis of PC using CA19.9 as a marker is associated with false positive and false negative rates [16–19]. Thus, future studies should be directed at investigating whether CA19.9 in combination with other markers can yield improved sensitivity and specificity [20].

S100A4 is a multifunctional Ca^2+^ signaling protein found in the cytoplasm and extracellular space, also known as metastasin (Mts1), pEL-98, CAPL, calvasculin, fibroblast-specific protein (FSP1), and so on [21–25]. S100A4 participates in a variety of biological functions, including inhibition of tumor cell apoptosis, promotion of cell proliferation and angiogenesis, and cell motility [26]. In addition, S100A4 may affect the expression and activity of matrix metalloproteinases, accelerate degradation of the basement membrane, and promote neovascularization, which inhibits the adhesion of tumor cells and increases their motility [27]. Recently, other molecules, including S100A4, have been reported as tumor markers for diagnosing PC [11, 28]. These tumor markers, which are expected to overcome the limitations of using CA19.9 as a prognostic factor, are frequently measured in patients with resectable PC.

However, so far, to the best of our knowledge, no studies have reported the relationship between the expression of S100A4 and the serum level of CA19.9 in PC and whether they have a combined effect on patient survival. In the current study, we simultaneously compared the expression level of S100A4 and serum level of CA19.9 in PC and investigated the effect of their interaction in the development of PC and their potential as prognostic factors.

## Methods

### Patients and tissue samples

We retrieved the medical records of patients that had undergone surgery and received a histopathological diagnosis for PC at the General Hospital of Xinjiang Military Region (*n* = 63) and the Luo Yang Central Hospital Affiliated to Zheng Zhou University (*n* = 65) between June 2000 and December 2007. In the study, a total of 128 formalin-fixed and paraffin-embedded PC tissue samples were used. The patients from whom these samples were collected had not received radiotherapy or chemotherapy before the surgery. This study included only histologically confirmed cases. Patients who received chemotherapy or radiation therapy before surgery and patients with incomplete clinical data were excluded from the study. Medical records were used to check the serum CA19.9 levels 1 week before the operation, and the limit of CA19.9 normal reference value (35.00 KU/L) was divided into positive and negative values. Using the address and contact information details listed in the medical records, the patient or the patient’s relatives were contacted by telephone to obtain information about the patient’s survival or death and the date of death, if applicable. As of December 31, 2013, all patients were followed up by telephone to obtain survival data. The median follow-up was 12 months (range 4–36 months). Given the retrospective nature of the study, the need for informed consent was waived and the study was approved by the Ethics Committee of Luo Yang Central Hospital Affiliated to Zheng Zhou University.

### Immunohistochemistry and scoring

Four-micrometer thick sections were cut from the paraffin-embedded tissue blocks obtained from 128 patients, mounted on slides, and incubated at 37 °C for 12 h. After the sections were deparaffinized with high concentrations of alcohol and xylene, they were incubated in a trypsin solution at 37 °C for 10 min to repair the antigen and then left for cooling at 20–30 °C. Two to three drops of 3% hydrogen peroxide solution were then added on the sections to block endogenous peroxidase activity. Sections were then incubated for 10 min at 37 °C. Subsequently, 2–3 drops of normal non-immune goat serum were added to the sections to block non-specific antigen binding and sections were then incubated at 37 °C for 10 min. Next, a 1/50 dilution of rabbit S100A4 antibody (ZA-0257, Beijing Zsbio Co. Ltd., People’s Republic of China) was added onto the sections and they were incubated for 2 h at 37 °C. Two to three drops of Polymer Helper reagent (PV-9000, Beijing Zsbio Co. Ltd., People’s Republic of China) were then added to the sections, and they were incubated at 37 °C for 20 min. Subsequently, 2–3 drops of polyperoxidase-anti-mouse/rabbit IgG (PV-9000, Beijing Zsbio Co. Ltd., People’s Republic of China) were added to the sections, and they were incubated at 37 °C for 20 min. The sections were washed in phosphate-buffered saline washing solution for 3–5 min between each of these steps. Sections were finally visualized using DAB, counterstained with hematoxylin, mounted in neutral gum, and analyzed using bright-field microscopy. Both positive and negative immunohistochemistry controls were routinely used. S100A4 positive slides from reagent company (Beijing Zsbio Co. Ltd., People’s Republic of China) were used as the positive control, and slides treated with PBS instead of the primary antibody were used as the negative control. Manufacturer’s instructions were followed to perform the empirical procedure.

The sections were reviewed in a blinded manner, and semi-quantitative analysis of the intensity and extent of immunostaining was performed. The distribution of staining for S100A4 was evaluated by the secondary scoring method with the percentage scores of 0: < 5%, 1: 5–25%, 2: 26–50%, 3: 51–75%, and 4: > 75% and staining intensity scores of 1: buff, 2: buffy, and 3: puce. When the product of two scores was greater than or equal to 1, the section was considered to be positive for S100A4 staining.

### Statistical analysis

The SPSS17.0 software (SPSS, Inc., Chicago, Illinois, USA) was used for statistical analyses. Association of categorical parameters was tested using chi-square test. Survival analysis was performed using the Kaplan–Meier method, and survival curve comparison between groups was performed using the log-rank test. Correlation analysis was performed using the Spearman correlation. The Cox proportional hazards model was used to perform multivariate analysis for all parameters significant in the univariate analysis. A bilateral *P* < 0.05 was considered statistically significant.

## Results

### Expression of S100A4 and serum levels of CA19.9 in PC

**Fig. 1:**
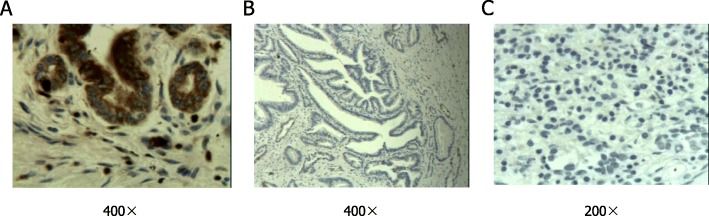
Expression of S100A4 in PC tissues and normal adjacent tissues. **a** Positive expression in PC. **b** Negative expression in PC. **c** Negative expression in normal adjacent tissues

Immunohistochemical analysis of S100A4 expression was performed on PC tissues. The positive reaction of S100A4 was observed as brown areas. S100A4 is mainly located in the cytoplasm, and a small amount is located in the nucleus or the membrane (Fig. 1a). Among the 96 (75.0%) of the 128 patients with PC whose tissues showed positive S100A4 expression, negative expression of S100A4 was found in 25% (32/128). No expression of S100A4 was observed in adjacent normal tissues (Fig. 1c). Higher serum CA19.9 levels (35 U/mL) were found in 89.1% (114/128) of patients with PC, whereas 10.9 % of patients (14/128) had levels lower than 35 U/mL.

### Expression of S100A4 and serum levels of CA19.9 and their relationships with the clinicopathological features of PC

The chi-square analysis showed that the positive expression of S100A4 was associated with tumor size (*P* = 0.028), differentiation (*P* = 0.007), TNM stage (*P* = 0.000), and lymph node metastasis (*P* = 0.025). Furthermore, positive expression of CA19.9 was associated with differentiation (*P* = 0.000) and lymph node metastasis (*P* = 0.003) (Table 1).

**Table 1:** Association between the expression of S100A4 and serum CA19.9 level and clinicopathological characteristics in PC *n* (%)

Clinicopathological parameter	Cases (*n*)	S100A4 expression		*P* value	Serum CA19.9 level		*P* value
		Positive (+)	Negative (−)		High (+)	Normal (−)	
Gender				0.917			0.489
Male	75	56 (58.3)	19 (59.4)		68 (59.6)	7 (50.0)	
Female	53	40 (41.7)	13 (40.6)		46 (40.4)	7 (50.0)	
Age (year)				0.355			0.203
< 60	57	45 (46.9)	12 (37.5)		53 (46.5)	4 (28.6)	
≥ 60	71	51 (53.1)	20 (62.5)		61 (53.5)	10 (71.4)	
Tumor size (cm)				0.028			0.699
≤ 2	5	3 (3.1)	2 (6.3)		5 (4.4)	0	
2–4	83	57 (59.4)	26 (81.3)		74 (64.9)	9 (64.3)	
>4	40	36 (37.5)	4 (12.5)		35 (30.7)	5 (35.7)	
Tumor location				0.217			0.521
Head	72	51 (53.1)	21 (65.6)		63 (55.3)	9 (64.3)	
Body/tail	56	45 (46.9)	11 (34.4)		51 (44.7)	5 (35.7)	
Differentiation				0.007			0.000
High	36	22 (22.9)	14 (43.8)		26 (22.8)	10 (71.4)	
Middle	66	49 (51.0)	17 (53.1)		62 (54.4)	4 (28.6)	
Low	26	25 (26.0)	1 (3.1)		26 (22.8)	0	
TNM stage				0.000			0.248
I	11	6 (6.3)	5 (15.6)		10 (8.8)	1 (7.1)	
II	47	26 (27.1)	21 (65.6)		39 (34.2)	8 (57.1)	
III	52	46 (47.9)	6 (18.8)		47 (41.2)	5 (35.7)	
IV	18	18 (18.8)	0		18 (15.8)	0	
Distant metastasis				0.076			0.194
Present	26	23 (24.0)	3 (9.4)		25 (21.9)	1 (7.1)	
Absent	102	73 (76.0)	29 (90.6)		89 (78.1)	13 (92.9)	
Lymph node metastasis				0.025			0.003
Present	66	55 (57.3)	11 (34.4)		64 (56.1)	2 (14.3)	
Absent	62	41 (42.7)	21 (65.6)		50 (43.9)	12 (85.7)	

### Correlation between S100A4 expression and serum CA19.9 level in PC

**Fig. 2:**
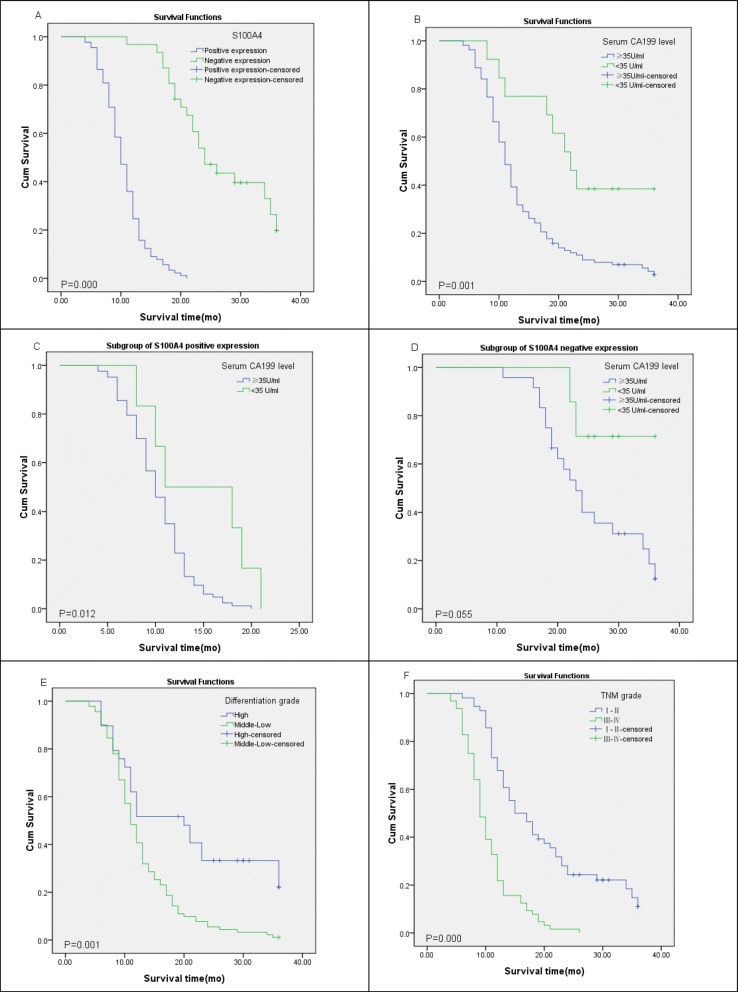
Kaplan-Meier survival curves of the patients with PC expression of S100A4 and serum levels of CA19.9. **a** Patients with positive expression of S100A4 vs negative expression of S100A4. **b** Patients with high CA19.9 level (≥ 35 U/ml) vs normal CA19.9 level (< 35 U/ml). **c** Subgroup of patients with positive expression of S100A4 and high CA19.9 level vs normal CA19.9 level. **d** Subgroup of patients with negative expression of S100A4 and high CA19.9 level vs normal CA19.9 level. **e** Patients with high differentiation vs moderate and low differentiation. **f** Patients with stages I–II vs stages III–IV

Of the 128 PC cases, 89 (69.5%) patients exhibited positive expression of S100A4 and high CA19.9 levels, and 7 (5.5%) cases showed negative expression of S100A4 and normal CA19.9 levels. Additionally, there were 32 (25.0%) cases with either negative expression of S100A4 or normal CA19.9 levels. Spearman’s correlation was used to examine the relationship between S100A4 expression and serum CA19.9 levels in PC; the results showed that S100A4 protein expression was positively correlated with serum CA19.9 levels (*ρ* = 0.202, *P* = 0.022) (Table 2). Meanwhile, the co-expression of both proteins correlated significantly with tumor differentiation (*ρ* = − 0.280, *P* = 0.001), TNM stage (*ρ* = − 0.389, *P* = 0.000), and lymph node metastasis (*ρ* = 0.254, *P* = 0.008) (Table 3). The co-expression of S100A4 and CA19.9 in patients with stage III–IV PC was higher than that in patients with stage I–II PC. Moreover, the co-expression of both proteins in the highly differentiated group was lower than that in the moderately and low-differentiated group (Table 3); the same was true in the lymph node metastatic group, which was related to survival and prognosis of patients (Fig. 2e, f).

**Table 2:** Correlation between S100A4 and CA19.9

	Serum CA19.9 level			
S100A4 expression	≥ 35 U/ml	< 35 U/ml	*ρ* value	*P* value
Positive (*n* = 96)	89	7		
Negative (*n* = 32)	25	7		
Total	114	14	0.202	0.022

**Table 3:** Relation between protein co-expression and clinicopathological parameters *n* (%)

Parameter	Case	S100A4 expression/serum CA19.9 level				*ρ*	*P* value
		(+)/(+)	(+)/(−)	(−)/(+)	(−)/(−)		
Differentiation						− 0.280	0.001
High	36 (28.1)	18 (14.1)	4 (3.1)	8 (6.3)	6 (4.7)		
Middle-low	92 (71.9)	71 (55.5)	3 (2.3)	17 (13.3)	1 (0.8)		
TNM stage						− 0.389	0.000
I–II	58 (45.3)	30 (23.4)	2 (1.6)	19 (14.8)	7 (5.5)		
III–IV	70 (54.7)	59 (46.1)	5 (3.9)	6 (4.7)	0 (0)		
Lymph node metastasis						0.254	0.008
Present	66 (51.6)	53 (41.4)	2 (1.6)	11 (8.6)	0 (0)		
Absent	62 (48.4)	36 (28.1)	5 (3.9)	14 (10.9)	7 (5.5)		
Case	128 (100)	89 (69.5)	7 (5.5)	25 (19.5)	7 (5.5)

### Positive S100A4 expression and high CA19.9 level in patients with PC are associated with poor overall survival

Notably, patients positive for S100A4 expression exhibited a significantly worse overall survival than those negative for S100A4 (*P* = 0.000) (Fig. 2a). Similarly, the overall survival in the group exhibiting high CA19.9 levels was significantly worse than in the group with the normal CA19.9 levels (*P* = 0.001) (Fig. 2b). Moreover, the prognosis of subgroups with positive S100A4 expression and high CA19.9 levels was significantly lower than patients with normal CA19.9 levels (*P* = 0.012) (Fig. 2c). However, in the subgroup of patients negative for S100A4 expression and with normal CA19.9 level, the prognosis remained unaffected (*P* = 0.055) (Fig. 2d). The univariate analysis was performed to assess the risk factors associated with the prognosis in patients with PC. As shown in Table 4, differentiation, TNM stage, distant metastasis, lymph node metastasis, positive S100A4 expression, and serum CA19.9 level were correlated with the overall survival of patients with PC. The S100A4(−) group had a 1-year cumulative survival rate of 96.8% and a median survival period of 24 months, both of which were higher than those in the S100A4(+) group (24.7%, 10 months, respectively). The 1-year cumulative survival rate (76.9%) and median survival period (21 months) of patients with serum CA19.9 level < 35 U/ml were higher than those in patients with serum CA19.9 level ≥ 35 U/ml (39.3%, 11 months, respectively); S100A4(−)/serum CA19.9 level < 35 U/ml group had a significantly higher survival rate than other groups. In addition (*P* = 0.000), a serum CA19.9 level of ≥ 35 U/mL was also identified as a significant independent predictor of worse overall survival (*P* = 0.001). Additionally, patients with PC having positive S100A4 expression and serum CA19.9 level of ≥ 35 U/mL showed a significant decrease in overall survival (*P* = 0.000) (Table 4). However, the overall survival was not associated with age, gender, tumor size, and tumor location. Furthermore, the multivariate analysis showed that differentiation, TNM stage, S100A4 expression, and serum CA19.9 levels were significant independent prognostic factors for overall survival of PC patients (Table 5).

**Table 4:** Univariate analyses of overall survival in patients with PC

Clinical parameter	Follow-up cases (*n*)	Median survival (months)	1-year cumulative survival rate (%)	HR(95%CI)	*P* value
Differentiation					0.000
High	29	12	51.7	4.351 (11.473–28.527)	
Middle	65	13	56.9	0.501 (12.018–13.482)	
Low	26	8	0	0.630 (6.766–9.234)	
TNM stage					0.000
I	11	15	72.7	1.651 (11.763–18.237)	
II	45	15	66.7	2.789 (11.534–22.466)	
III	46	10	26.1	0.751 (8.529–11.471)	
IV	18	8	11.1	1.061 (5.921–10.079)	
Distant metastasis					0.004
Present	25	9	24	0.389 (8.238–9.762)	
Absent	95	12	48.4	0.696 (10.636–13.364)	
Lymph node metastasis				–	0.000
Present	61	10	29.5	0.554 (8.914–11.086)	
Absent	59	13	57.6	1.280 (10.491–15.509)	
S100A4					0.000
Positive	89	10	24.7	0.471 (9.077–10.923)	
Negative	31	23	96.8	2.127 (19.831–28.169)	
Serum CA19.9 level					0.001
≥ 35 U/ml	107	11	39.3	0.517 (9.987–12.013)	
< 35 U/ml	13	21	76.9	2.397 (17.303–26.697)	
S100A4/CA19.9					0.000
S100A4(+)/CA19.9(+)	83	10	22.9	0.504(9.012–10.988)	
S100A4(+)/CA19.9(−)	6	11	50.0	4.899(1.398–20.602)	
S100A4(−)/CA19.9(+)	24	22	95.8	1.752(19.567–26.433)	
S100A4(−)/CA19.9(−)	7	25	100.0		

*S100A4(+/−)* positive/negative expression of S100A4, *CA19.9(+/−)* higher serum CA19.9 levels (≥ 35 U/mL)/normal CA19.9 level (< 35 U/ml)

**Table 5:** Multivariate Cox regression analysis of patients with PC

	*n*	*B*	SE	Wald	df	Sig.	Exp(B)	95.0% CI	
Differentiation									
(high/middle/low)	36/66/26	0.991	0.200	24.459	1	0.000	2.693	1.819	3.988
TNM stage									
(NM stage	11/47/52/18	0.490	0.123	15.923	1	0.000	1.632	1.283	2.075
S100A4 expression									
(positive/negative)	96/32	− 2.448	0.399	37.696	1	0.000	0.086	0.040	0.189
Serum CA19.9 level									
(high/normal)	114/14	− 0.859	0.394	4.751	1	0.029	0.424	0.196	0.917

## Discussion

An increasing number of studies have revealed that S100A4 performs functions related to tumor growth and metastasis, and S100A4 is also overexpressed in many tumor tissues [29, 30]. However, so far, only few studies have reported the relationship between S100A4 and PC, and even these studies have included sample sizes not exceeding 100 specimens [31, 32]. Tsukamoto et al. [11] showed that S100A4 is highly expressed in PC and is strongly associated with tumor invasion, which can predict a poor prognosis in PC patients. Ai et al. [33] found that the expression of S100A4 in PC is significantly correlated with tumor diameter, TNM stage, and poor prognosis, but not with differentiation, lymph node metastasis, and distant metastasis. Furthermore, S100A4 was implicated in the process of metastasis in PC and strongly linked to neoplasm invasion, metastasis, and unfavorable prognosis in patients, but had no obvious relationship with lymph node metastasis [34]. In contrast, it has also been reported that S100A4 is associated with tumor differentiation, but not with lymph node metastasis and TNM stage [35].

In this study, we examined the expression of S100A4 in 128 PC tissues using immunocytochemistry and confirmed its correlation with clinicopathological parameters and prognosis. Our results showed that the positive expression rate of S100A4 in low/intermediate differentiation groups was significantly higher than that in the high differentiation group. In addition, the expression levels of S100A4 were found to be considerably related to tumor size, tumor differentiation, TNM stage, and lymphatic invasion in PC patients. S100A4 expression in PC tissues may be an independent prognostic risk factor; the prognostic risk in patients with high expression of S100A4 was twice than in patients with low expression of S100A4 (Table 4). Thus, measurement of S100A4 expression levels is useful for predicting the prognosis of patients with PC. However, no relation was found between S100A4 expression and distant metastasis. This may have been caused by the small sample size and needs to be further studied.

We also compared the expression level of S100A4 and serum level of CA19.9 in PC tissues and investigated their interaction in the development of PC and their effects on prognosis. Among the 128 patients for whom preoperative serum CA19.9 levels were available, higher serum CA19.9 levels (≥ 35 U/mL) were found in 89.1% of patients. Meanwhile, high serum CA19.9 levels were found to be significantly related to tumor differentiation (*P* = 0.000) and lymph node metastasis (*P* = 0.003). Here, we further demonstrated that high serum CA19.9 levels were associated with poor prognosis in PC patients. Previous studies have shown that CA19.9 not only plays a role as a tumor marker in PC but also plays an important biological function in the occurrence and development of PC. Galli et al. showed that PC cells are more prone to certain metabolic variations or defects, which promote the production of CA19.9 and Lewis-a antigens, thus enabling these cancer cells to gain the advantages of growth, invasion, and metastasis, which further promotes the growth of PC cells [36]. In more than one study, CA19.9 and Lewis-a antigens have been shown to play a tumor-promoting role in PC [37, 38]. Furthermore, Lewis-b antigen overexpression has been shown to inhibit invasion, adhesion, and metastasis of PC cells. In the current study, we explored the relationship between S100A4 and CA19.9 in PC and found a significant correlation between them using Spearman’s test. To our knowledge, this is the first study to determine the association between tissue S100A4 expression and the pre-operative serum level of CA19.9 in PC. As the results from the Spearman’s test showed a highly significant positive correlation between S100A4 and CA19.9, we investigated the relationship between protein co-expression and clinicopathological parameters. We found that the co-expression of both proteins was significantly correlated with TNM stage, tumor differentiation, and lymph node metastasis. Furthermore, analysis using the Kaplan-Meier method revealed that the S100A4(−)/serum CA19.9 level (< 35 U/ml) group had a higher 1-year cumulative survival rate and median survival period than that of the other groups. Additionally, positive expression of S100A4 and higher serum CA19.9 level (≥ 35 U/mL) contributed to a significant decrease in overall survival in PC patients. Therefore, we hypothesized that CA19.9 is likely to also play an important role in the development and progression of pancreatic malignant tumors; S100A4 interacted with CA19.9 to promote invasion and metastasis of PC.

In addition, in this study, the co-expression of S100A4 and CA19.9 in patients with stage III–IV PC was higher than that in patients with stage I–II PC, while that in PC patients in the highly differentiated group was lower than that in the moderately and low-differentiated group. The same result was observed in the lymph node metastatic group, which was related to survival and prognosis of PC patients, further supporting the argument that analysis for CA19.9 in combination with S100A4 could better determine the prognosis of PC patients.

Though the study has produced some credible results, the study had a few limitations. The study cohort had a relatively small number of samples, and the follow-up time to assess patient survival was relatively short. Thus, further studies are needed to confirm these findings and determine the role of S100A4 and CA19.9 as reliable clinical predictors of PC outcomes.

## Conclusions

In summary, S100A4 and CA19.9 were overexpressed in PC patients and played important roles in PC transformation. Furthermore, we demonstrated, for the first time, that the expression levels of S100A4 and CA19.9 were positively correlated. Thus, S100A4 and CA19.9 can be used as biomarkers to evaluate PC prognosis. Analysis for CA19.9 in combination with S100A4 can yield improved sensitivity and specificity, compensating for the limitations associated with using CA19.9 for the evaluation of resection in PC patients.

## Data Availability

The analyzed data sets generated during the study are available from the corresponding author on a reasonable request.

## Abbreviations

CA19.9(+/−): Higher serum CA19.9 levels ( 35U/mL)/normal CA19.9 level (< 35U/ml)
PC: Pancreatic carcinoma
S100A4: Calcium-binding protein S100A4
S100A4(+/−): Positive/negative expression of S100A4
